# Vincristine-Induced Cranial Neuropathy

**Published:** 2014

**Authors:** Ahmad TALEBIAN, Razieh Moazam GOUDARZI, Mahdi MOHAMMADZADEH, Azadeh Sadat MIRZADEH

**Affiliations:** 1 Trauma Research Center, Kashan University of Medical Sciences, Kashan, Iran; 2 Department of Pediatrics, Kashan University of Medical Sciences, Kashan, Iran

**Keywords:** Vincristine, Side effect, Wilms’ tumor, Ptosis

## Abstract

Vincristine (VCR) is a vinca alkaloid that is used for treatment of many malignancies. The vinca alkaloids are neurotoxic, usually causing a peripheral neuropathy, but cranial neuropathies are rare as side effects. Described here is the case of a 2.5-year-old boy, a known case of Wilms’ tumor, treated by vincristine (0.067 mg/kg/day) and dactinomycin (0.045 mg/kg/day) after surgery. Three weeks after treatment, he presented with bilateral ptosis. Neurological examination revealed bilateral ptosis with normal pupillary reflex and eye movement. He received 3.015 mg cumulative dose of vincristine before development of ptosis.

Treatment with pyridoxine (150 mg/m2 p.o. BID) and pyridostigmine (3 mg/kg p.o. BID) was started as neuroprotective agents, and after 7 days the problem disappeared. The treatment continued for 6 weeks and there were no signs of ptosis or a recurrence in follow up 2 months later.

## Introduction

Vincristine (VCR) is a vinca alkaloid used for treatment of many malignancies such as acute lymphoblastic leukemia, neuroblastoma, Ewing’s sarcoma, Wilms’ tumor, rhabdomyosarcoma, Hodgkin’s disease, non-Hodgkin’s lymphoma (1), idiopathic thrombocytopenic purpura, and autoimmune hemolytic anemia (2).

Neurotoxicity is a well-known side effect of VCR that was first reported in 1967. VCR results in axonal degeneration and delay in distal axon transportation. Since VCR has low central nervous system (CNS) penetration, the neurological side effects frequently manifest in the peripheral nervous system (3).

The standard dose of VCR is 1-2 mg/m2 once every 1-3 weeks and consumption of doses over 2 mg is neurotoxic, especially as a weekly prescription.Therefore, multiple regimes are needed to decrease the neurotaxic effect (1). 

Vincristine induced neurotoxicity can be divided into four groups:

1. Peripheral neuropathy

2. Autonomic neuropathy

3. Encephalopathy

4. Cranial neuropathy

The most common side effect is dose-dependent peripheral neuropathy with early depression of the deep-tendon reflexes. Other known side effects are paresthesia, gait disorder, cranial nerve palsies, and brain dysfunction in most advanced cases (4).

## Case Report

A 2.5-year-old boy with a known case of Wilms’ tumor was treated by VCR (0/067 mg/kg/day) and dactinomycin (0/045 mg/kg/day) after surgery. Three weeks after treatment, he presented with bilateral ptosis. There was no companying symptom in the patient history.

**Fig 1:**
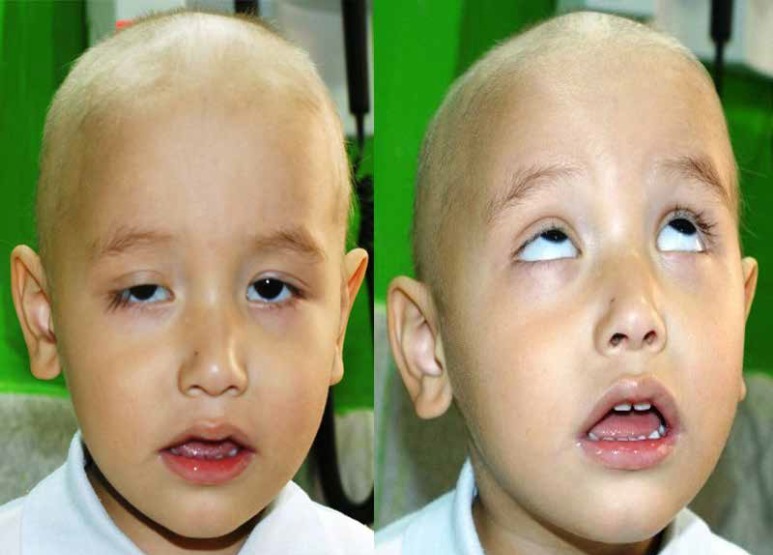
Physical exam showed the patient had bilateral ptosis with normal pupil reflex and eye movement

There was no trauma, recent eye surgery, or neurological disease in the patient medical history. Past drug history included only chemotherapy drugs. There were no positive points in the family history as well. There was nothing found in the physical exam, except for bilateral ptosis. Pupil reflex and eye movement were normal (fig1).

Laboratory data such as, LFT, CBC, and electrolytes were normal.

We had suspicions of a brain lesion and an MRI was ordered, but the MRI and the ensuing lumbar puncture were also normal.

Because the etiology of ptosis was unknown in evaluations and it is known as a side effect from VCR treatment, treatment by pyridoxine (150 mg/m2 p.o.BID), and pyridostigmine (3 mg/kg p.o.BID) were started as neuroprotective agents. After 7 days the problem disappeared. 

The treatment continued for 6 weeks and there were no signs of ptosis or a recurrence in follow up visits over the next 2 months.

## Discussion

Vincristine (VCR) is a vinca alkaloid and neurotoxicity is a well-known side effect of VCR (3).

Signs of VCR induced neuropathy usually appear in 2-19 weeks after treatment (5). The clinical manifestation of VCR induced neurological complications are paresthesia, ataxia, gait disorder, wrist and foot drop, depression of DTR, facial nerve palsy, weakness, optic neuropathy (1), transient cortical blindness, ptosis, abdominal colic pain, constipation, urinary retention, orthostatic hypotension, jaw pain, hoarseness, and loss of sensory neural hearing (6).

The incident rate of these complications is dependent on age, VCR dose, treatment duration, nutritional condition, liver function, history of peripheral neuropathy, and simultaneous consumption of drugs like Methotrexate, L-asparginas, allopurinol, erythromycin, INH, mitomycin-C, phenytoin, or itraconazole (5). Treatment by VCR may result in encephalopathy, seizure, or SIADH. Consumption of 5-6 mg VCR in most patients reveals early signs of toxicity, but remarkable toxicity is not seen in cumulative doses less than 15-20 mg (6). 

The definitive diagnosis of VCR induced neuropathy is related to the exclusion of other etiologies that cause similar clinical features. 

Findings in the present case that support a diagnosis of VCR induced neuropathy are:

1- the time course of ptosis after treatment;

2- the absence of pathologic findings in CSF analysis and MRI;

3- The resolution of ptosis after treatment.

There are no entirely convincing reports of effective pharmacologic measures to prevent or to treat vincristine induced neuropathy, apart from a few case reports of cranial neuropathy that was treated with pyridoxine and pyridostigmine (2).

Whether the VCR-induced neuropathy is recovered by pyridoxine is still unknown. 

For the first time, pyridoxine was used for Isoniazidinduced neuropathy treatment in tuberculosis patients. Isoniazid (INH) neuropathy results from pyridoxine consumption and its deficiencies (3). 

Neuroprotective pyridoxine effects on VCR-induced neuropathy in animal models showed promise, but its useful effects on humans remain unproven (3).

In a study of 24 patients suffering from stage II breast cancer under adjuvant chemotherapy with VCR, 1.5 gm of pyridoxine was administrated daily for 6 weeks, but treatment was not effective. However, the high-dose of pyridoxine used in this study has previously been reported to cause neuropathy by itself in the absence of VCR, which casts doubt as to the etiology of the neurological symptoms and the negative results (3).

In a case report published in a leukemia research journal, a 21-year-old patient suffering from ALL, after afflicted 4 times of chemotherapy with dysphagia, dysarthria, and an inability to open the mouth wide enough to eat. A physical exam showed bilateral 7 and 12 nerve palsy and loss of tongue movement. Up to that point, a cumulative dose of VCR in the patient was 2mg /m2 (actual dose: 28mg). Finally, after exclusion of other causes, patient was diagnosed with VCR-induced neuropathy and was treated with pyridoxine 150mg/m2 daily(total dose 250mg), after 5 days the symptoms disappeared and after 2 weeks there was a complete recovery.

**Table 1:** Summary of case reports of pyridoxine ± pyridostigmine for VCR- induced neuropathy

**Author**	**Age** **(years)**	**Diagnosis**	**Symptoms**	**Cumulative dose of VCR**	**Treatment**	**Recovery time**
Bay et al.	5 Female	ALL	Cranial neuropathy	6mg/m2 (actual 3.8 mg)	Pyridoxine 300mg/m2/day Pyridostigmine 6mg/kg/day	7 days (Complete Recovery: 14 days)
Ozyurek et al (7)	4 Male	ALL	Cranial neuropathy + peripheral neuropathy	4.5mg/m2 (actual unknown)	Pyridoxine 150mg/m2/day Pyridostigmine 3mg/kg/day	14 days (Complete Recovery: 28 days)
Duman et al.(8)	2 Male	Wilms	Cranial neuropathy + peripheral neuropathy	9.8mg/m2 (actual 8.8 mg)	Pyridoxine 150mg/m2/day	5 days (Complete Recovery: 20 days)
Müller et al.(9)	2 Male	Sarcoma	Cranial neuropathy	19.5mg/m2 (actual 11.7 mg	Pyridoxine 300mg/m2/day Pyridostigmine 6mg/kg/day	7 days (Complete Recovery: 21 days)
Dejan et al(10)	5Male	ALL	Cranial neuropathy	10.5mg/m2 (actual 10.5 mg)	Pyridoxine 150mg/m2/day Pyridostigmine 3mg/kg/day	14 days (Complete Recovery: 28 days)

Table 1 indicates other case reports regarding VCRinduced neuropathy with successful treatments of pyridoxine and prydostigmine (Table 1)(5,7-10). 

Another case report was published in the Indian Journal of Pediatrics, in which a 5-year-old girl was described who suffered from ALL and was under chemotherapy treatment with VCR, L-asparaginase, prednisolone, and daunorubicin. Five days after the fourth dose of VCR was administered, bilateral ptosis appeared. Further surveys were in favor of VCR-induced neuropathy in this patient as well.

The patient was treated with pyridoxine (150 mg/ m2.p.o BID) and pyridostigmine (3 mg/ kg.P.O BID) and she recovered after 7 days and completely recovered after 2 weeks (5).

According to the aforementioned reports and patients, it seems that the application of pyridoxine and pyridostigmine can be successfully used in patients with VCR-induced neuropathy.
